# Chorea-ballism as a dominant clinical manifestation in heteroplasmic mitochondrial encephalopathy, lactic acidosis, and stroke-like episodes syndrome with A3251G mutation in mitochondrial genome: a case report

**DOI:** 10.1186/s13256-018-1936-0

**Published:** 2019-03-06

**Authors:** Durjoy Lahiri, Vishal Madhukar Sawale, Subhadeep Banerjee, Souvik Dubey, Biman Kanti Roy, Shyamal Kumar Das

**Affiliations:** Department of Neurology, Bangur Institute of Neurosciences, IPGMER and SSKM Hospital, Kolkata, 700025 India

**Keywords:** Mitochondrial encephalopathy, lactic acidosis, and stroke-like episodes (MELAS), Chorea, Ballism, A3251G mutation

## Abstract

**Background:**

Mitochondrial encephalopathy, lactic acidosis, and stroke-like episodes, the most common maternally inherited mitochondrial disease, can present with a wide range of neurological manifestations including both central and peripheral nervous system involvement. The most frequent genetic mutation reported in mitochondrial encephalopathy, lactic acidosis, and stroke-like episodes syndrome is A3243G in *MT-TL1* gene. Stroke-like episodes, dementia, epilepsy, lactic acidemia, myopathy, recurrent headaches, hearing impairment, diabetes, and short stature constitute the known presentations in this syndrome. Among the abnormal involuntary movements in mitochondrial encephalopathy, lactic acidosis, and stroke-like episodes syndrome, myoclonus is the commonest. Other movement disorders, including chorea, are rarely reported in this disorder.

**Case presentation:**

A 14-year-old South Asian boy from rural Bengal (India), born of a second degree consanguineous marriage, with normal birth and development history, presented with abnormal brief jerky movements involving his trunk and limbs, with recurrent falls for 10 months. We present here a case of heteroplasmic mitochondrial encephalopathy, lactic acidosis, and stroke-like episodes syndrome with A3251G mutation, in which the clinical picture was dominated by a host of involuntary abnormal movements including chorea-ballism, myoclonus, and oromandibular dystonia in a backdrop of cognitive decline, seizure, and stroke-like episode. A final diagnosis was established by muscle biopsy and genetic study. Haloperidol was administered to control the involuntary movements along with introduction of co-enzyme Q, besides symptomatic management for his focal seizures. Six months into follow-up his seizures and abnormal movements were controlled significantly with slight improvement of cognitive abilities.

**Conclusion:**

The dominance of hyperkinetic movements in the clinical scenario and the finding of a point mutation A3251G in *MT-TL1* gene make this a rare presentation.

## Background

Mitochondrial cytopathies are characterized by energy failure in the cellular system. These disorders are associated with an increasingly large number of heterogeneous clinical presentations. Mitochondrial encephalopathy, lactic acidosis, and stroke-like episodes (MELAS) is the most common maternally inherited mitochondrial disease, with broad manifestations including stroke-like episodes, dementia, epilepsy, lactic acidemia, myopathy, recurrent headaches, hearing impairment, diabetes, and short stature. Multiple genetic mutations have been found in association with MELAS syndrome [1]. An A>G mutation at position 3243 in the *MT-TL1* gene of the mitochondrial DNA (mtDNA)-encoding mitochondrial transfer RNA (tRNA)^Leu(UUR)^ is responsible in more than 80% of cases. Additional uncommon mutations (for example, m.3271T>C and m.3251A>G) in the *MT-TL1* gene have also been reported to cause MELAS syndrome. Impaired mitochondrial translation and protein synthesis including the mitochondrial electron transport chain complex subunits form the basis of failed energy production by mitochondria [2].

Myoclonus is the most frequent abnormal movement that has been reported to occur in MELAS [3, 4]. Rare cases of other movement disorders including dystonia [4] and chorea [5, 6] are also available in the literature. However, a combination of multiple abnormal movements in a single patient with MELAS is hard to find. Moreover, in most of these reported cases the A3243G mutation was detected.

We present here a case of heteroplasmic MELAS syndrome with A3251G mutation, in which the clinical picture was dominated by a host of involuntary abnormal movements including chorea-ballism, myoclonus, and oromandibular dystonia in a backdrop of cognitive decline, seizure, and stroke-like episode.

## Case presentation

A 14-year-old South Asian boy from rural Bengal (India), born of a second degree consanguineous marriage, with normal birth and development history, presented with abnormal brief jerky movements involving his trunk and limbs, with recurrent falls for 10 months. The jerks were neither stimulus sensitive nor present during sleep. No loss of consciousness was reported to occur with these jerky movements. Recurrent convulsions involving the left half of his body, without impairment of awareness, was present for 8 months. It was followed by insidious onset of mild weakness of the left half of his body for 7 months. Subsequently he suffered progressive decline in his general ability to maintain average daily activity independently for 5 months. He had to discontinue schooling because of his failing cognitive functions. For 2 months prior to presenting to us, he developed rapid dance-like movements involving all four limbs that flowed from one muscle to the other in a more or less continuous fashion. Occasionally it would become somewhat flinging particularly in his upper limbs. There was no history of similar illness in the family. He received all the scheduled vaccines as was stated by his mother.

The height of the boy was 150 cm and he did not have any dysmorphic facial features. A clinical examination revealed generalized choreiform movements as the most obvious finding. These movements intermittently became flinging in nature, resembling ballism. Generalized myoclonic jerks were seen embedded inside the flurry of chorea-ballism. When he was asked to protrude his tongue, besides motor impersistence, oromandibular dystonia was also found. He had severe dysarthria with apparently preserved comprehension. A limited cognitive assessment revealed reduced attention span as well as short-term memory impairment. Rigidity was obvious in all four limbs along with dystonia in both lower limbs. Weakness in the left half of his body along with brisk reflexes and extensor plantar on left side was also detected on motor system evaluation.

**Fig. 1:**
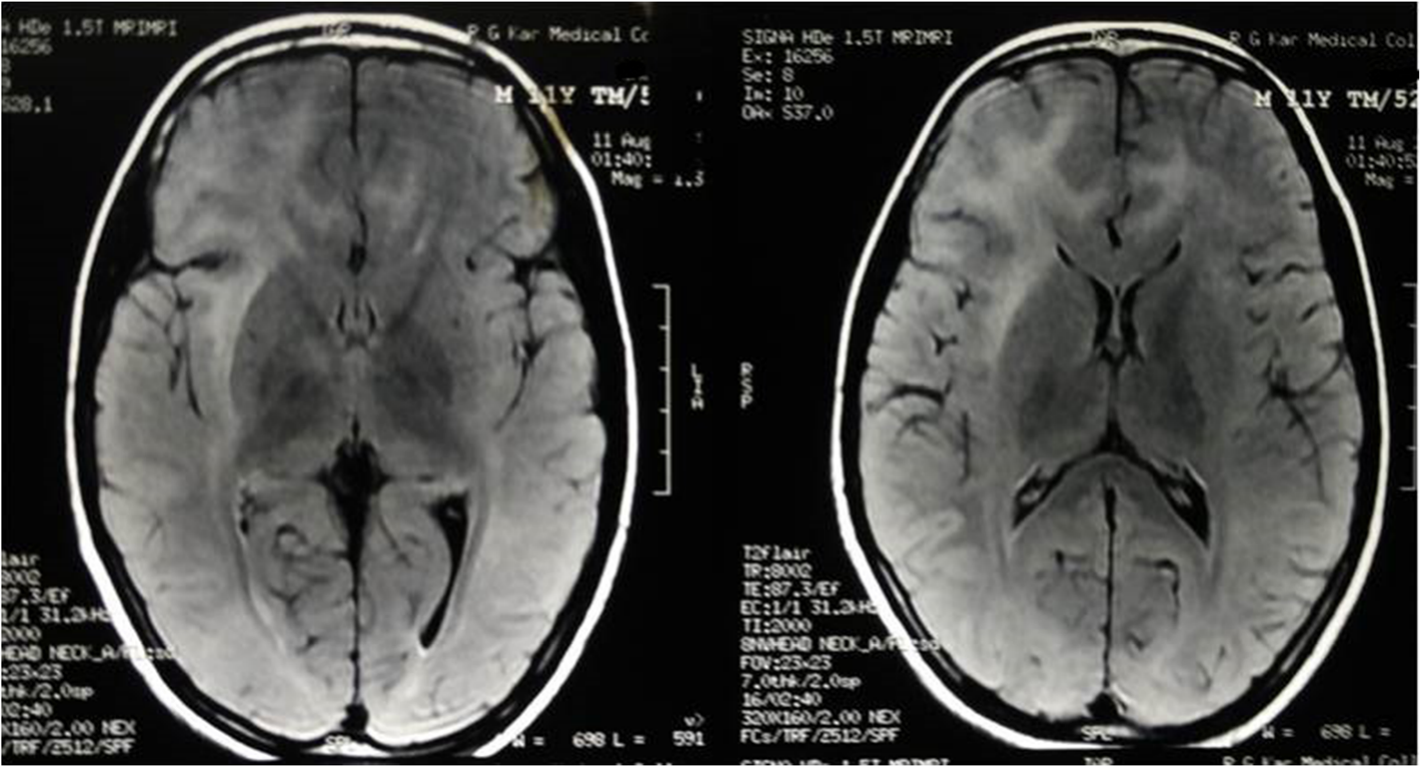
Magnetic resonance imaging of the brain (T2 fluid-attenuated inversion recovery sequence) shows white matter hyperintensities in both frontoparietal regions, more on the right side

**Fig. 2:**
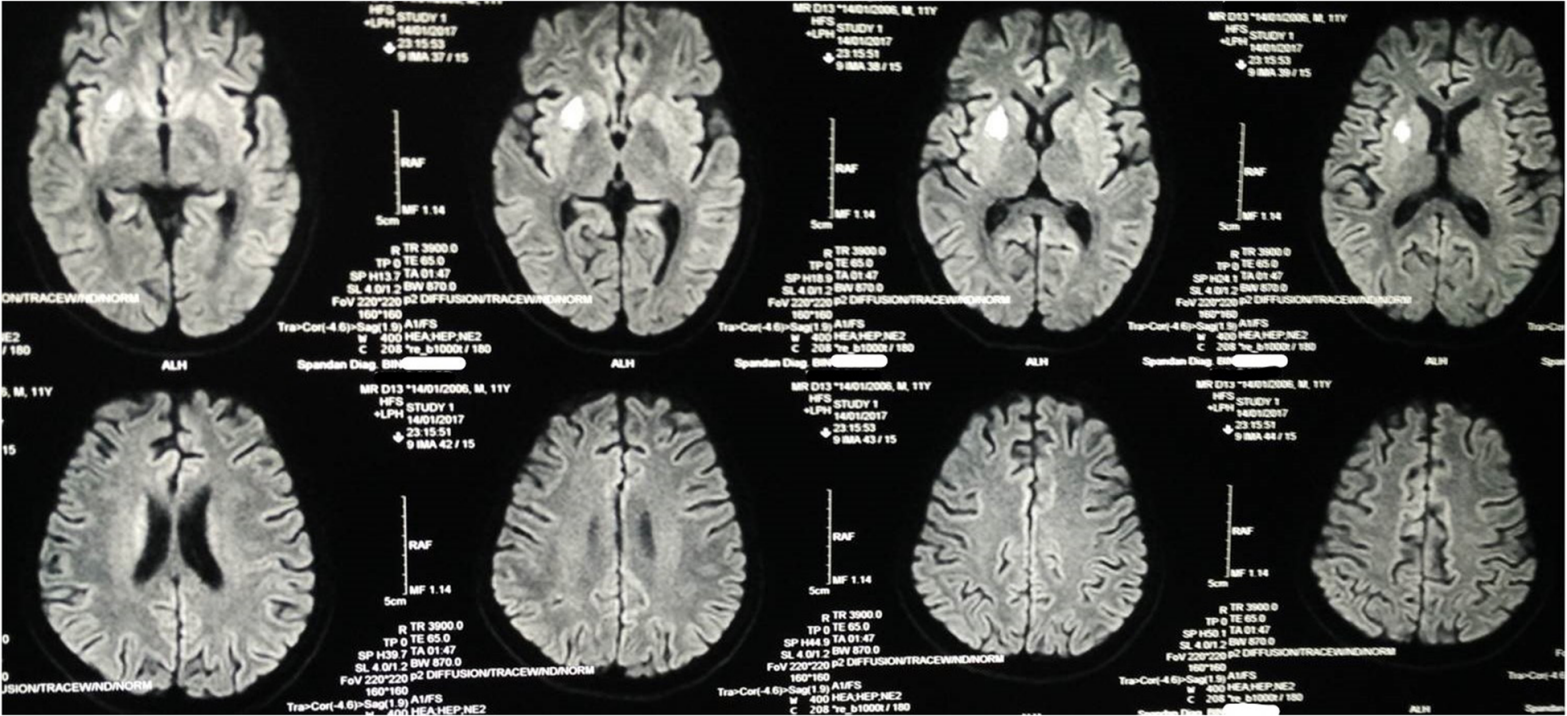
Magnetic resonance imaging of the brain (diffusion-weighted sequence) shows a small area of diffusion restriction in the capsule-ganglionic area

**Fig. 3:**
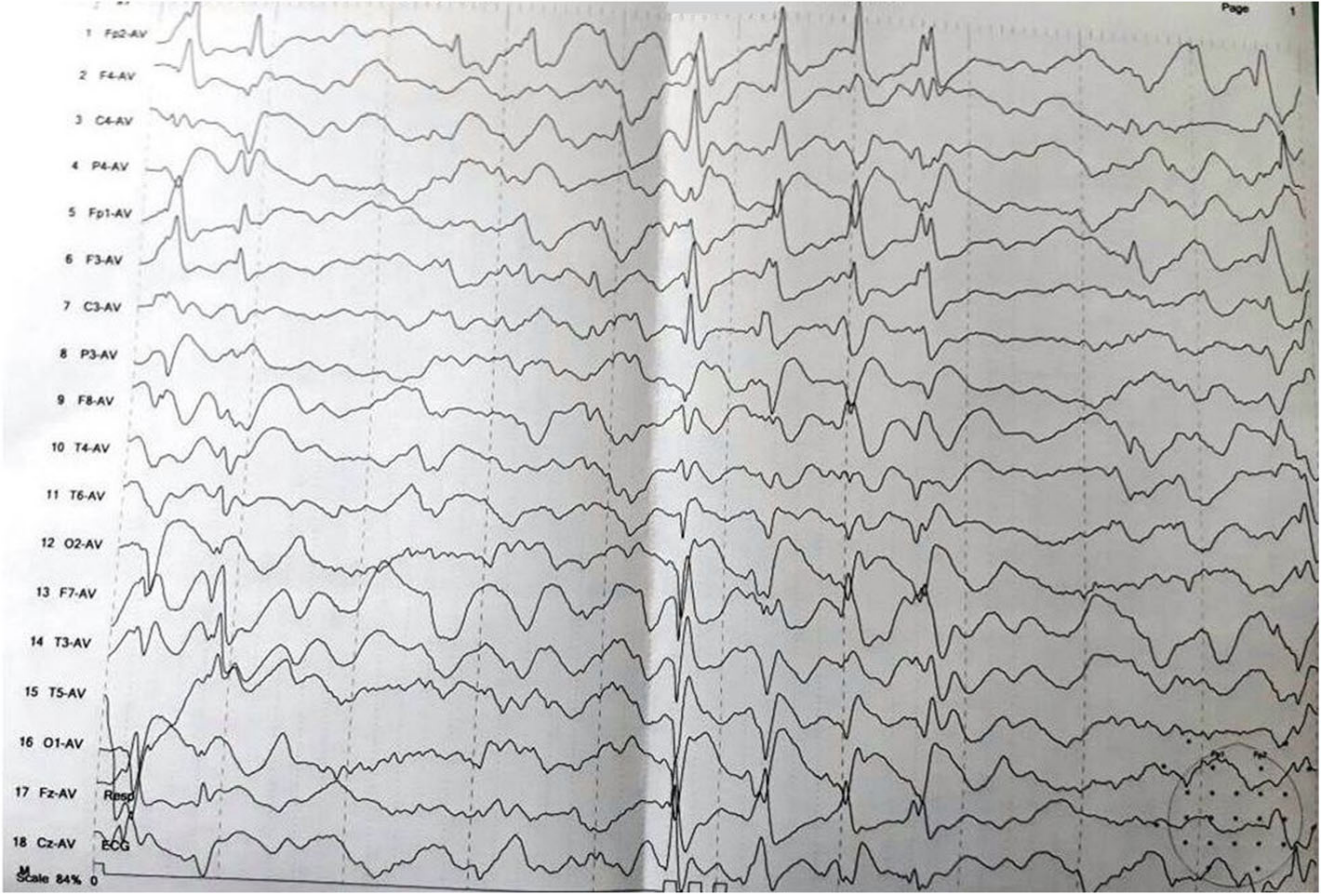
Electroencephalogram (average montage) shows spike wave discharges arising from both frontal areas, more amplitude on right side

**Fig. 4:**
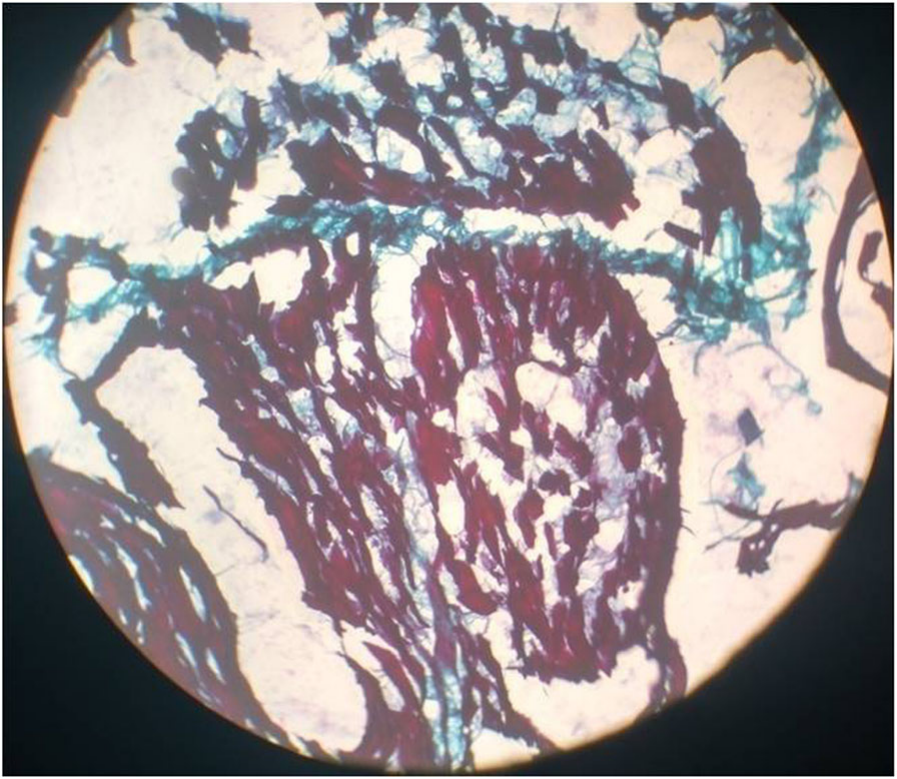
Microscopic examination of frozen section biopsy in modified Gömöri trichrome stain reveals ragged red fibers that constitute 40% of all the muscle fibers examined

Routine laboratory parameters revealed impaired fasting glucose (120 mg/dl), mildly raised liver enzymes and creatine phosphokinase (CPK) level of 820 IU/L. Other blood and urine parameters were within normal limits. Screening investigation for Wilson’s disease, storage disorders, and metabolic disorders were all negative. A routine cerebrospinal fluid (CSF) study was unremarkable and anti-measles antibody was negative. Anti-nuclear antibody in blood was also negative. His serum level of lactate was 36 mg/dl (2–19 mg/dl) while CSF lactate was 42 mg/dl. Shortening of PR interval (0.10 second) was found in electrocardiography. Two-dimensional echocardiography was devoid of any abnormality. Serial brain imaging was done at different centers throughout the course of his illness. On studying his MRI brain images sequentially, a relapsing remitting pattern of lesions was detected. On T2/fluid-attenuated inversion recovery sequence (FLAIR) there were hyperintense lesions that mainly involved subcortical white matter in frontoparietal areas (Fig. 1). An area of diffusion restriction was found in the right capsule-ganglionic region (Fig. 2) that temporally coincided with the onset of left hemiconvulsions and hemiparesis. Magnetic resonance spectroscopy (MRS), done at our center, showed the presence of lactate peak in brain lesions. Brainstem auditory response revealed bilateral prolonged latency. Electromyography (EMG) showed short duration low-amplitude polyphasic motor unit action potential which was suggestive of myopathic pattern. Spike-wave discharges were observed arising from bilateral frontal areas on electroencephalography (Fig. 3). A muscle biopsy, which was done from left vastus lateralis, revealed ragged red fibers (Fig. 4), suggestive of mitochondrial failure and deposition of abnormal mitochondria below the plasma membrane of muscle fibers.

According to the clinical criteria, MELAS syndrome was the most probable diagnosis in our case and we needed to confirm the diagnosis. As a facility for analysis of respiratory chain enzymes in the muscle was not available, we decided to search for underlying genetic abnormality in mtDNA. A polymerase chain reaction (PCR) method was employed for this purpose. Amplification of DNA in whole blood sample of our patient was performed for detection of mutations 3243A>G, 3271T>C, and 3251A>G in mitochondrial tRNA leucine 1(MT-TL1), by using appropriate wild type and mutant type specific primers for each and a common reverse primer for all. Genetic analysis result was as following: A>G point mutation at position 3251 of *MT-TL1* gene of the mtDNA with heteroplasmy of 70%.

After reaching the diagnosis, valproate was taken off and lamotrigine was introduced. He was put on co-enzyme Q supplement and haloperidol for abnormal movements. Six months into follow-up his seizures and abnormal movements were controlled significantly with slight improvement of cognitive abilities.

## Discussion

A combination of central and peripheral nervous system involvement is the usual pattern in mitochondrial encephalomyopathies. MELAS syndrome, one of the most frequent maternally inherited mitochondrial disorders, was first delineated in 1984 [5]. The molecular basis of MELAS syndrome was initially discovered in 1990 when adenine to guanine transition at position 3243 of mtDNA (m.3243A>G) in the *MT-TL1* gene encoding tRNA^Leu(UUR)^ was found to be associated with this syndrome [2]. The diagnostic criteria for MELAS was first established in 1992 [3] and have been modified subsequently. The clinical diagnosis of this syndrome is based on the following three invariant criteria: (1) stroke-like episodes before age of 40 years, (2) encephalopathy characterized by seizures and/or dementia, and (3) mitochondrial myopathy evident by lactic acidosis and/or ragged red fibers. The diagnosis is confirmed if there are also at least two of the following criteria: (1) normal early psychomotor development, (2) recurrent headaches, and (3) recurrent vomiting episodes. The MELAS Study Group committee in Japan published the latest diagnostic criteria by which diagnosis is considered definitive with at least two category A criteria (headaches with vomiting, seizures, hemiplegia, cortical blindness, and acute focal lesions in neuroimaging) and two category B criteria (high plasma or CSF lactate, mitochondrial abnormalities in muscle biopsy, and a MELAS-related gene mutation) [7]. In our case, the clues to the diagnosis were: high CSF lactate, relapsing remitting brain lesion with lactate peak in MRS, and finally the presence of ragged red fibers in muscle biopsy. The genetic study, which was performed to confirm diagnosis, revealed an uncommon point mutation: A3251G.

When it comes to abnormal movements, myoclonus has been the commonest (10–30%) to be reported in MELAS [3, 4], but it is rarely the predominant or presenting symptom. Other involuntary movements are genuinely rare in this syndrome. Of the 36 patients with the A3243G mutation studied by Hammans *et al*. [4], only one developed dystonia, a few years after the onset of stroke-like episodes and seizures. Writer’s cramp as a presenting symptom was reported in one patient with A3243G mutation [8]. Acute-onset chorea, triggered by hyperglycemia, has been reported in two cases with the A3243G mutation [5, 6]. The case of one patient with rare akinetic-rigid parkinsonism was reported, due to a 4-base pair deletion in the mitochondrial cytochrome *b* gene [9]. Orofacial dyskinesia and parkinsonism with predominant gait freezing in a case of MELAS was reported from Thailand [10]. However, A3251G mutation was never reported in these case studies. Our case was unusual in the sense that our patient had an array of abnormal movements that included chorea-ballism, myoclonus, and dystonia. Moreover, the clinical presentation was dominated by generalized choreiform movements, which in itself is a rare occurrence in MELAS. Obviously, hyperglycemia was not the cause of chorea in the index case; rather direct involvement of basal ganglia by T2/FLAIR hyperintense lesions seems to be a plausible explanation here.

Another interesting aspect of our case was the presence of subtle myopathy which was evidenced by raised CPK level, myopathic pattern in EMG, and ragged red fibers in histology. This case exemplifies the fact that even in the face of dominant central nervous system (CNS) involvement, subclinical signs of peripheral nervous system (PNS) damage should be sought particularly if mitochondrial cytopathy is a consideration.

## Conclusion

We report this case of MELAS syndrome which revealed a rare point mutation and a rare combination of multiple involuntary movements as the dominant clinical presentation.

## Abbreviations

CPK: Creatine phosphokinase
CSF: Cerebrospinal fluid
EMG: Electromyography
FLAIR: Fluid-attenuated inversion recovery sequence
MELAS: Mitochondrial encephalopathy, lactic acidosis, and stroke-like episodes
MRS: Magnetic resonance spectroscopy
mtDNA: Mitochondrial DNA
tRNA: Transfer RNA
